# Chemical Composition of Milk and Rumen Microbiome Diversity of Yak, Impacting by Herbage Grown at Different Phenological Periods on the Qinghai-Tibet Plateau

**DOI:** 10.3390/ani10061030

**Published:** 2020-06-13

**Authors:** Qingshan Fan, Metha Wanapat, Fujiang Hou

**Affiliations:** 1 State Key Laboratory of Grassland Agro-Ecosystems, Key Laboratory of Grassland Livestock Industry Innovation, Ministry of Agriculture and Rural Affairs, College of Pastoral Agriculture Science and Technology, Lanzhou University, Lanzhou 730020, Gansu, China; Fanqsh18@lzu.edu.cn; 2 Tropical Feed Resources Research and Development Center (TROFREC), Department of Animal Science, Faculty of Agriculture, Khon Kaen University, Khon Kaen 40002, Thailand; metha@kku.ac.th

**Keywords:** chemical composition, 16S rRNA gene sequencing, rumen bacteria, yak milk, herbage

## Abstract

**Simple Summary:**

Native values of herbage grown at different phenological periods and rumen diversity of microbial population would impact rumen fermentation end-products and milk compositions of yaks (*Bos grunniens*). The research was conducted in 12 female yaks grazing on the Qinghai-Tibet Plateau (QTP). The results revealed that the phenological periods (VS: Vegetative stage, May; BS: Bloom stage, August; SS: Senescent stage, December) significantly influenced the nutritive values of herbages, microbial diversity and, as a consequence affected on the yak milk yield and compositions. We concluded that the observed differences resulted from the combined effects of phenological periods, herbage composition, and herbage availability. The findings of this study were of great value and useful for current understandings and onwards to conduct further research and for possible practical implementation for the yak cows grazing on QTP.

**Abstract:**

To estimate how native herbage of three different phenological periods modify rumen performance and milk quality of yak grazing alpine meadow. In this study, milk composition and the diversity of the rumen microbial community were measured in 12 full-grazing female yaks on the Qinghai-Tibet Plateau (QTP). The nutrient composition of three phenological periods was determined: Vegetative stage (VS), bloom stage (BS), and senescent stage (SS). High-throughput sequencing of the bacterial 16S rRNA gene was used. The results showed that crude protein (CP) content of herbage in BS was higher than that in vs. and SS (*p* < 0.05), and neutral detergent fiber (NDF) content of herbage in SS was higher than that in vs. and BS (*p* < 0.05). Milk solids and fat contents were higher in the vs. and SS than in BS (*p* < 0.05). However, milk protein content was higher for the vs. and BS than those for SS (*p* < 0.05). The total volatile fatty acid (VFA), acetate, and propionate concentrations were higher in vs. and BS than in SS (*p* < 0.05). The community richness estimates (Chao1 estimator) of vs. were higher than that in BS and the SS (*p* < 0.05). The diversity indices (Shannon index) of the BS were higher than that vs. and the SS (*p* < 0.05). Spearman correlation analysis between the milk composition, ruminal fermentation parameters, and the relative abundances of the rumen bacteria showed that milk protein content, total VFA, acetate, and propionate concentrations were positively correlated with the relative abundances of the genera *Desulfovibrio*, *Prevotella_1*, and *Butyrivibrio_2* and was negatively correlated with *Olsenella*, *Ruminococcaceae_UCG.010*, and *Rikenellaceae_RC9_gut_group* abundances. Collectively, the results revealed that there were significant differences in nutrient composition of herbage, chemical composition of yak milk, and microbial diversity in rumen at different phenological stages. The correlations between ruminal fermentation parameters, chemical constituents of yak milk, and some genera of ruminal bacteria might be indicative that the ruminal fermentation parameters and chemical constituents of yak milk are strongly influenced by the rumen bacterial community composition.

## 1. Introduction

Yaks (*Bos grunniens*) have exhibited the ability as the only bovine to adapt and thrive in an extremely harsh environment (low humidity, temperature, oxygen levels, strong winds, and UV radiation) and high-altitude level of the Qinghai-Tibet Plateau (QTP), at 2000–5000 m above sea level. The total population of yaks is estimated at 14.2 million and about 13.3 million are raised in China [1]. Yak is an important nutrient contributing to milk, meat, hair, and cheese to the people living in the QTP. Natural alpine meadows in the QTP also have qualities of low temperature, high altitude, high variability in temperature and precipitation, and these factors directly affect plant productivity and nutrition [2]. The growth period of grass is approximately 100 to 150 day per year, and the dormant period lasts for about 7 months. Grasses begin to regreen each year in April, reach their peak biomass in August, and wither in November. Yaks commonly graze on natural occurring pastures all year round without receiving nutritional supplements. Because of the long cold season, plateau grassland is withered, and the most critical situation is heavy snow disaster, in which the land is covered by thick snow and yaks cannot access any herbage. This induces dramatic body weight reduction and mortality. Previous research showed that once food is available again in the short-term warm season, yaks are in a compensatory growth status with higher growth rates and feed efficiency [3]. After long-time evolution, yaks obtained the unique physiological traits to adapt to the extreme high-altitude environment.

Based on the fact that the yak represents the primary source of milk for the people of this region, it is imperative to understand the genetic makeup of the yak and how this can influence milk biosynthesis. Yak milk is more nutritive to dairy cow milk in nutrient composition, especially protein and fat [4], and contains higher fat content of 5.5 to 7.5% as compared to 3.50% in bovine milk and higher protein content of about 4.9–5.9% compared to 3.14% in bovine milk [5]. Seasonal variation of feeds especially during the cold season greatly influence traditional yak farming [6]. Supplements are not a common practice even under the low protein content of pastures [5]. Therefore, determining composition of the milk of yak, impacting by herbage grown at different phenological periods, is essential to potentially enhance milk yield and quality.

The rumen microbiomes are of prime importance to sustaining good rumen ecology, health, and ruminant productivity. Researchers in ruminant nutrition showed an understanding of the pivotal role of the rumen in digesting fibrous feeds and providing essential nutrients to the host animal [7]. Feeding ruminants with fibrous feeds will be able to provide humans with foods, mainly milk and meat from nonhuman-edible herbage. The major population of the rumen microbiome has been identified. Bacteria, which usually comprise most of the species richness, are widely persistent geographically across multiple ruminant species and individual animals [8], and many microbiomes can be considered symbiotic with ruminants, as they provide metabolic activities and products essential for the host. There is a good understanding that variation in the ruminal microbial community is responsible for the differences in the efficacy and efficiency at which feed is converted to ruminant products. Previous studies have shown that factors such as diet, age, species, and seasons all impact ruminal microbes, and diet is a particularly important contributor [9,10]. Diet and environmental conditions (temperature and precipitation) influence the microbial community composition [11,12]; however, little is known about the bacterial community changes over time when ruminants are on similar diets, especially in the QTP where the herbage differs considerably throughout a whole year due to the harshness of the environment.

The objectives of the current study were to: (1) Investigate the effects of different phenological periods on chemical composition of the milk, ruminal fermentation parameters, and rumen microbial diversity of free-range yaks; (2) determine the relationship between herbage nutrient content, rumen microbiome diversity, and milk composition of yaks. The results are excepted to help find ways to increase the nutritive value of yak milk, provide green and healthy products, and provide scientific data for the development of yak milk and their products by suitable supplementary feeding during the dry season. We hypothesized that under similar conditions covering physiological characteristics, different phenological periods would significantly affect the chemical constituents of milk and rumen microbial diversity of yaks.

## 2. Materials and Methods

### 2.1. Ethics Statement

The animal sampling protocol and management were in accordance with the rules and regulations of experimental field management protocols (file No: 2010-1 and 2010-2), which were approved by Lanzhou University.

### 2.2. Experimental Design

The research was conducted at Maqu Grassland Agricultural Trial Station, Maqu County, Gansu Province, China (33°40′4′′ N, 101°52′12′′ E; elevation 3704 m), on the northeastern edge of the QTP. It is cataloged as plateau continental climate. Annual mean temperature was 2.0 °C, with monthly average temperature, ranges from a high of 10–15 °C in July and August to below −5 °C in December and January; mean annual precipitation was 602 mm, of which some two-thirds typically falls in the months May–August. The abundance of solar energy is evidenced by the number of annual sunshine hours of 2583.9 h and the herbage grass growth period is 150–168 day. The cumulative ≥0 °C temperature is 1240.3 °C and the experimentally studied duration was 8 months (May 2018 to December 2018). Data for long-term mean climate variables were obtained from the Gannan meteorological station. Twelve healthy five-year-old QTP yaks with an average weight of 246.53 ± 13.28 kg were selected, sequentially numbered, and allowed to graze. These twelve yaks, without supplementary feeding during the experimental stage, were allowed access to water. Ruminal fluid and milk were collected during the following herbage growth stage: Vegetative stage (VS, 15 May), bloom stage (BS, 15 August), and senescent stage (SS, 15 December), and alpine meadow samples were collected during the same three stages. The dominant species were *Kobresia graminifolia*, *K. capillifolia*, *K. humilis*, *K. tibetica*, *Elymus nutans*, *Potentilla anserina L*, *Stipa aliena*, and *Festuca ovina*, and proportions of the main herbage on three phenological periods are illustrated in Table 1. Biomass was highest in August at 286.5 g/m^2^ and the ratio of high-quality herbage to the total above-ground biomass was 56.96%. Yaks year-continuously graze the alpine meadow.

The high-quality herbage refers to the grasses which are classified as sedges and grasses, while the other herbage refers to the grasses which are not classified as sedges and grasses.

### 2.3. Sampling of Milk and Measurements of Chemical Composition

Each of the phenological periods during the experiment included a 7-day sampling period. In the three 7-day periods, the yaks were hand milked once daily at 09:00 h, and milk yield was recorded. The volume of milk consumed by calves during the daytime was not considered. About 400 mg milk was sampled at each milking and stored at −20 °C for later analysis.

For milk samples (36 samples), nitrogen content was chemically analyzed by the Kjeldahl method, ether extract by Soxhlet extraction with diethyl ether for fat content [13]. Crude protein was calculated as N × 6.38 for milk [5]. Lactose content of milk was measured using the Lane-Eynon method [14]. Components of milk were expressed as g 100 g^−1^ milk dry matter (DM).

### 2.4. Sampling of Plant and Measurements of Chemical Composition

Herbage samples were collected by quadrats (50 cm × 50 cm) from grass on which the animals grazed during the vegetative stage (15 May 2018), the bloom stage (15 August 2018), and the senescent stage (15 December 2018). Twenty quadrats (50 cm × 50 cm), of which the distance between plots exceeded 12 m, were randomly placed in the alpine meadow to collect grass samples and cut out the ground part of the quadrats with scissors at three sample collection times. Samples from each quadrat were used to investigate dominant species, dried in a 60 °C oven for 24 h to constant weight at the laboratory, and were ground in a mill and passed through a 1-mm sieve for further analysis.

Dry matter (DM), crude protein (CP), and crude fat ether extract (EE) were measured using Association of Official Analytical Chemists (AOAC) methods [13]. The concentration of neutral detergent fiber (NDF) and acid detergent fiber (ADF) in 60 samples (20 samples per period) were analyzed using an Ankom 2000 fiber analyzer (Ankom Technology, Fairport, NY, USA) according to the methods described by [15].

### 2.5. Sampling of Rumen Contents and Measurement of Fermentation Variables

The rumen contents (36 samples) were collected using an oral stomach tube [16]. This method has been used in previous studies [16,17], and this method was used extensively and the rumen fluid was collected successfully without contamination. The first 100 mL of rumen fluid was discarded to avoid reticulum fluid or salivary contaminated fluid or body surface bacteria, and then 50 mL of rumen fluid was collected from each animal prior to grazing in the morning, and immediately measured by pH meter (HI 9024C; HANNA Instruments, Woonsocket, RI, USA). The rumen fluid samples were thoroughly filtered with four layers of cheesecloth and divided for three portions to be analyzed for volatile fatty acids (VFA), NH_3_-N concentration, and for DNA extraction. The volatile fatty acids (VFA) concentrations in ruminal fluid were analyzed using gas chromatography-mass spectrometry (GC-MS522; Wufeng Instruments, Shanghai, China). The ammonia-nitrogen (NH_3_-N) concentrations were measured as described by [18].

### 2.6. Deoxyribonucleic Acid (DNA) Extraction, Amplification, and Sequencing

Total DNA was extracted using the repeated bead beating plus column method [19]. This method uses cell lysis by bead beating in the presence of high concentrations of SDS, salt, and EDTA. The subsequent DNA was purified using a QIAamp DNA Stool Mini Kit (Qiagen, Hilden, Germany). The quality and quantity of the DNA samples were measured using the NanoDrop 2000 spectrophotometer (NanoDrop Technologies, Wilmington, DE, USA). The amplicon DNA was amplified using the 338F/806R primer set (5′-ACTCCTACGGGAGGCAGCAG-3′/5′-GGACTACHVGGGTWTCTAAT-3′) [20], where the barcode is an eight-base sequence unique to each sample. The V3-V4 regions of the bacterial 16S ribosomal RNA gene were amplified by PCR (94 °C for 5 min, followed by 28 cycles at 94 °C for 30 s, 55 °C for 30 s, and 72 °C for 60 s and a final extension at 72 °C for 7 min). PCR reactions were analyzed using triplicate 25 μL reactions containing 30 ng DNA template, 1 μL of each primer (5 μM), 3 μL BSA (2 ng/μL), 12.5 μL 2×Tap Plus Master Mix, and 7.5 μL double distilled H_2_O (dd H_2_O). The resulting PCR products were separated in a 2% agarose gel, and further purified using the AxyPrep DNA Gel Extraction kit (Axygen Biosciences, Union City, CA, USA). Purified amplicons were pooled in equimolar portions and subjected to paired-end sequencing with an Illumina MiSeq platform (Illumina, Inc., San Diego, CA, USA).

### 2.7. Sequencing Data Processing and Analysis

Pair-end reads were merged by FLASH [21]. Data acquired through high-throughput sequencing were processed using the QIIME (v 1.8.0 http://qiime.org/index.html), and bases with quality scores higher than 20 were retained for further analysis [22]. Operational taxonomic units were clustered with 97% similarity cutoff using UPARSE (version 7.1 http://drive5.com/uparse/) [23], and chimeric sequences were identified and removed using UCHIME [24]. The α diversity, based on OTU (operational taxonomic unit) tables rarified to the same sequencing depth, was calculated as a measure of the richness (Chao 1 and observed_species) and diversity (Shannon and PD_Whole_tree) of bacterial communities in all animal samples [25]. These sequence data were submitted to the NCBI database under accession number PRJNA560628.

### 2.8. Statistical Analysis

Chemical composition of herbage, the rumen fermentation end-products of yaks, composition of yak milk, and the quantification of total bacteria were statistically analyzed using a completely randomized design with one-way analysis of variance (ANOVA) (version 9.2, SAS Institute Inc., Cary, NC, USA). Duncan’s method for multiple comparisons was used for variables where the treatment effect was significant (*p* < 0.05), and a significant tendency was based on 0.05 ≤ *p* < 0.10. Spearman correlation analysis between chemical composition of herbage, bacterial (genus), and ruminal fermentation parameters was performed using R corrplot.

## 3. Results

### 3.1. Nutritive Value of Yak Milk and Nutrient Compositions of Herbage

The OM content in the herbage did not differ among the phenological periods (Table 2). The CP and fat contents were higher for the BS than those for the vs. and BS (*p* < 0.05). Fibrous fractious (NDF and ADF) contents were higher for the SS than those in the vs. and BS (*p* < 0.05). Total solids and fat content of the yak milk were higher for the vs. and SS than those for the BS (*p* < 0.05) (Table 3). The protein content was higher for the vs. and BS than those for the SS (*p* < 0.05). The milk yield and lactose content were higher for the BS than those for the vs. and SS (*p* < 0.05).

### 3.2. Rumen Volatile Fatty Acid Composition

The environmental parameters in the rumen showed different trends during different phenological periods (Table 4). The concentration of rumen NH_3_-N in BS was higher than that in vs. and SS (*p* < 0.05). The concentration of TVFA, acetate (C_2_), propionate (C_3_), butyrate (C_4_), and valerate (C_5_) were lower in SS than in vs. and BS (*p* < 0. 05). The concentration of isobutyrate was higher in BS than in vs. and SS (*p* < 0.05), and was higher in vs. than in SS (*p* < 0.05). The concentration of isovalerate (C_5_) in vs. was higher than in BS and SS, SS was higher than in BS (*p* < 0.05). The molar proportion of the propionate and isobutyrate concentration were higher in BS than vs. and SS (*p* < 0.05). The molar proportion of the isovalerate was higher in SS than in vs. and BS (*p* < 0.05).

### 3.3. Bacterial Community Diversity, Richness and OTUs

**Figure 1:**
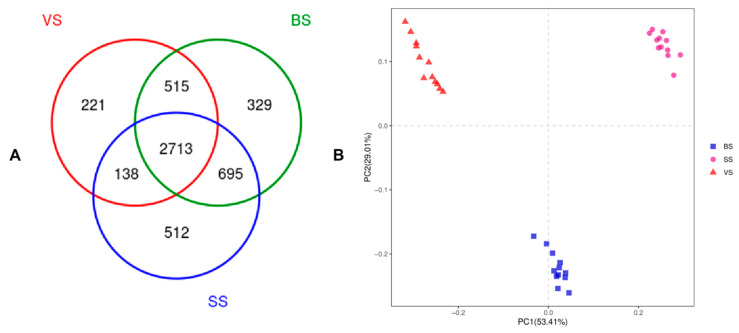
Differences in bacterial community richness and operational taxonomic units (OTUs) in different phenological periods. (**A**): Venn diagram showing the different and similar OTUs in different phenological periods; (**B**): Principal coordinate analysis (PCoA) of the yak ruminal microbiota in different phenological periods. The PCoA plots were constructed using the unweighted Unifrac method. VS: Vegetative stage, BS: Bloom stage, SS: Senescent stage.

**Figure 2:**
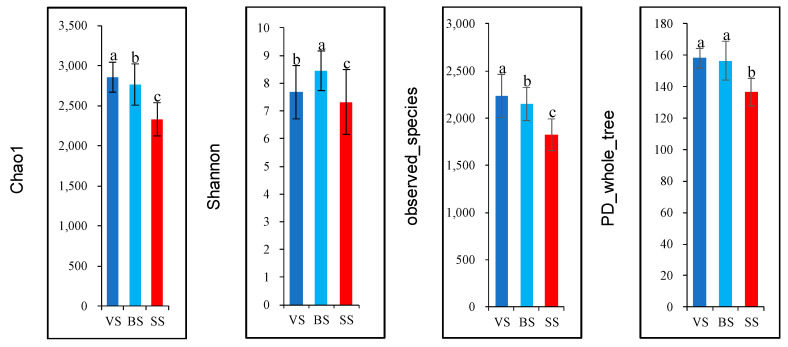
Alpha diversity indices of rumen bacteria in yak cows in different phenological periods. ^a–c^ Mean values with different superscripts are different at *p* < 0.05 according to Duncan’s multiple-range test. VS: Vegetative stage, BS: Bloom stage, SS: Senescent stage.

As shown in Figure 1A, 2713 OTU were shared in different phenological periods, the OTU numbers of the VS, BS, and the SS were 3587, and 4252, and 4058, respectively. The principal coordinate analysis showed clear separations of rumen bacteriome in different phenological periods yaks at the genus level based on the Bray–Curtis dis-similarity matrices (Figure 1B). The alpha diversity index analysis was presented in Figure 2. The community richness estimates (Chao 1 estimator) of the vs. was enhanced (*p* < 0.05), as compared with the BS and the SS (*p* < 0.05). The diversity indices (Shannon index) of the BS was enhanced (*p* < 0.05), as compared with the vs. and the SS (*p* < 0.05). The observed_species of the vs. was higher (*p* < 0.05), as compared with the BS and the SS (*p* < 0.05).

### 3.4. Rumen Bacterial Phylum and Genera

**Figure 3:**
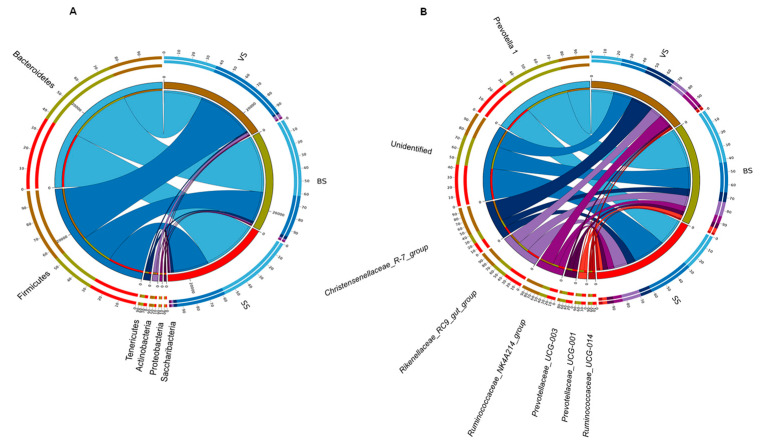
Bacterial phyla (**A**) and genus (**B**) (relative abundance >0.1% in at least 60% of the yaks within each phenological period) of 3 the phenological periods visualized using Circos. VS: Vegetative stage, BS: Bloom stage, SS: Senescent stage.

Eighteen phyla were found in the rumen fluid samples of yak. Among all the 18 phyla, the following phyla namely *Bacteroidetes*, *Firmicutes*, *Tenericutes*, *Actinobacteria*, *Synergistetes*, and *Proteobacteria* were detected as the predominant phyla (Table 5 and Figure 3A). The relative abundance of *Bacteroidetes* and *Actinobacteria* phylum were higher in the vs. than in the BS and SS (*p* < 0.05). The phylum *Firmicutes* and *Proteobacteria* were more abundant in the BS than in the vs. and SS (*p* < 0.05). The relative abundance of the phylum *Synergistetes* was higher in the SS than in the vs. and BS (*p* < 0.05). The phylum *Tenericutes* was more abundant in the BS and SS than in the vs. (*p* < 0.05). At the genera level, different phenological periods had an important effect on the relative abundance of the major genera (Table 5 and Figure 3B). The relative abundance of the genus *Prevotella_1* and *Rikenellaceae_RC9_gut_group* were more abundant (*p* < 0.05) in the SS than in the vs. and BS. The relative abundance of the genus *Prevotellaceae_UCG-001*, *Ruminococcaceae_UCG-005*, and *Senegalimassilia* were higher (*p* < 0.05) in the vs. than in the BS and SS, and the relative abundance of the genus *Christensenellaceae_R-7_group*, *Ruminococcaceae_NK4A214_group*, and *Butyrivibrio_2* were significantly higher (*p* < 0.05) in the BS than in the vs. and SS. The genus *Prevotellaceae_UCG-003* and *Ruminococcaceae_UCG-010* were more abundant (*p* < 0.05) in the SS than in the vs. and BS, respectively.

### 3.5. Milk Composition and Ruminal Fermentation Parameters in Relation to Nutrient Composition of Herbage

**Figure 4:**
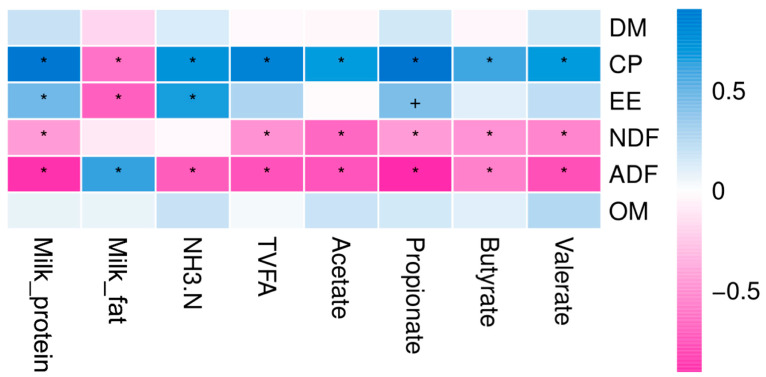
Spearman correlation and clustering analysis between milk composition, ruminal fermentation parameters, and nutrient composition of herbage. The depth of the color indicates the correlation between species and environmental factors. DM: dry matter, CP: crude protein, EE: ether extract, OM: organic matter, NDF: neutral detergent fiber, ADF: acid detergent fiber, TVFA: Total volatile fatty acids, NH3-N: ammonia nitrogen. The “+” and “*” indicates the different level at 0.05 and 0.01, respectively.

Correlation regression analysis was conducted to identify the correlations between milk composition, ruminal fermentation parameters, and the nutrients composition of herbages. The milk composition, ruminal fermentation parameters, and the nutrients composition of herbage were taken as correlated with each other (*p* < 0.05). The milk_protein and propionate concentration were positively correlated with the CP and EE in herbage and negatively correlated with the NDF and ADF in herbage (Figure 4). The milk_fat content was highly positively correlated with the ADF in herbage, while it was negatively correlated with the CP and EE in the herbage. The rumen ammonia-nitrogen concentration was positively correlated with the CP and EE in herbage and was negatively correlated with the ADF. Furthermore, total VFA, acetate, butyrate, and valerate population concentration were positively correlated with the CP of herbage, while it was negatively correlated with the NDF and ADF.

### 3.6. Milk Composition and Ruminal Fermentation Parameters in Relation to Main Bacteria at Genus Level

**Figure 5:**
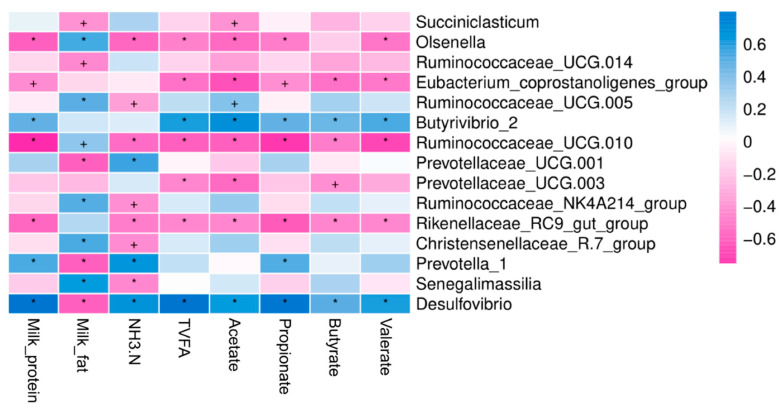
Spearman correlation and clustering analysis between milk composition, ruminal fermentation parameters, and main bacteria at genus level. The depth of the color indicates the correlation between species and environmental factors. TVFA: Total volatile fatty acids, NH_3_-N: ammonia nitrogen. The “+” and “*” indicates the different level at 0.05 and 0.01, respectively.

The analysis of the correlation regression was taken to determine the correlation between milk composition, fermentation parameters, and rumen bacterial population relative abundance. The milk composition, ruminal fermentation end products, and on the rumen bacteria relative abundance at the genus level were taken as closely correlated with each other (*p* < 0.05). The milk_protein content was positively correlated with the relative abundances of the genera *Desulfovibrio*, *Prevotella_1*, and *Butyrivibrio_2* and was negatively correlated with *Olsenella*, *Ruminococcaceae_UCG.010*, and *Rikenellaceae_RC9_gut_group* abundances (Figure 5). The milk_fat content was closely correlated with the relative abundances of the genera *Olsenella*, *Ruminococcaceae_UCG.005*, *Ruminococcaceae_UCG.010*, *Ruminococcaceae_NK4A214_group*, *Christensenellaceae_R.7_group*, and *Senegalimassilia* was negatively correlated with *Succiniclasticum*, *Ruminococcaceae_UCG-014*, *Prevotellaceae_UCG-001*, *Prevotella_1*, and *Desulfovibrio* abundances. The NH_3_-N concentration in the rumen was closely correlated with the relative abundances of the genera *Prevotellaceae_UCG-001*, *Prevotella_1*, and *Desulfovibrio* and was negatively correlated with *Olsenella*, *Ruminococcaceae_UCG.005*, *Ruminococcaceae_UCG.010*, *Ruminococcaceae_NK4A214_group*, *Rikenellaceae_RC9_gut_group*, *Christensenellaceae_R.7_group*, and *Senegalimassilia* abundances. Total fatty acids (VFA) production was closely correlated with the relative abundances of the genera *Desulfovibrio*, *Prevotella_1*, and *Butyrivibrio_2* and was negatively correlated with *Olsenella*, *Eubacterium_coprostanoligenes_group*, *Ruminococcaceae_UCG.010*, and *Rikenellaceae_RC9_gut_group* abundances. The acetate population was also closely correlated with the relative abundances of the genera *Ruminococcaceae_UCG.005*, *Butyrivibrio_2*, and *Desulfovibrio* and was negatively correlated with *Succiniclasticum*, *Olsenella*, *Eubacterium_coprostanoligenes_group*, *Ruminococcaceae_UCG.010*, *Prevotellaceae_UCG-003*, and *Rikenellaceae_RC9_gut_group* abundances. The butyrate (C_4_) and valerate (C_5_) molar were closely correlated with the related abundances of the genera *Butyrivibrio_2* while *Desulfovibrio* and was negatively correlated with *Olsenella*, *Eubacterium_coprostanoligenes_group*, *Ruminococcaceae_UCG.010*, and *Rikenellaceae_RC9_gut_group* abundances.

## 4. Discussion

There are many factors that could affect the quality of herbage, of which the species and the phenological period of herbage are important factors [26]. With the postponement of growth period, the content of CP in herbage gradually increased, and the content of NDF and ADF gradually decreased [27]. Results under this study revealed that the concentration of CP in the herbage in the BS was significantly higher than the vs. and the SS. The protein content of milk was increased for the vs. and BS than those for the SS. Since the yaks were allowed to graze under unsuitable environmental conditions, the milk quality varies with the phenological periods and with the climate changes [27]. This finding was in agreement with that of the increased protein quantity in the herbage could have resulted in the higher protein content of yak milk at the BS [28]. The NDF and ADF content was greater for the SS than those for the vs. and BS. The proportion of fat of the milk in the SS was significantly higher than the vs. and the BS. The enhanced crude fiber can offer more acetic acid and butyric acid for the production mammary gland to synthesize more fat, which may be the reason for the higher fat content of yak milk in the SS [29].

It is well illustrated that ruminal pH is an important factor for assessing fermentation in the rumen [30]. Rumen fluid pH decreased significantly with the decrease of dietary NDF level, and low NDF diet contained more easily fermented carbohydrates [31]. Rumen microbes could ferment rapidly and produce large amounts of VFA and organic acids, resulting in decreased ruminal pH. Our findings were in accordance with previous efforts. Propionate is the major substrate for gluconeogenesis in the lives for the ruminant hosts [32]. It is reported that acetic acid in the rumen can reduce the efficiency of energy utilization [33]. Under the vs. and SS the ratio of acetic/propionic acids was enhanced, implying that rumen fermentation mode changed and efficiency reduced, and BS had the lowest of the proportion of acetate and propionate and the highest TVFA level, implying that during the BS, the energy utilization efficiency was higher when compared with vs. and SS. Nitrogen in the diet is the main source of rumen NH_3_-N, and its concentration is affected by the degradation rate and absorption rate of nitrogen in the rumen, which can reflect the utilization of nitrogen by microorganisms [34,35]. The results obtained under this study indicated that the NH_3_-N content in the BS was significantly higher than in the vs. and SS, which may be due to the increase of non-structural carbohydrate/structural carbohydrate, the increase of dietary fermentable energy, the increase of easily degradable protein, the enhancement of microbial activity, and the increase of NH_3_-N content in the rumen [36]. An earlier study reported that ruminal NH_3_-N increased linearly in response to increasing dietary CP [37]. Under this study, the CP in herbage was closely correlated with the NH_3_-N concentration. In the rumen, VFA, mainly acetic (C_2_), propionic (C_3_), and butyric acids (C_4_) produced as microbial fermentation end-products, are a major source of absorbed energy and can account for 70–80% of the digestible energy intake in ruminants [38,39]. In this study, the concentration of TVFA was significantly lower in SS than in vs. and BS. Higher concentrations of VFA in rumen at BS suggests that herbage at BS can provide more energy to the yaks than those grown at the vs. and SS. Ruminal TVFA increased linearly in response to increasing dietary CP [36]. Under this current study, TVFA was closely correlated with the CP and was negatively correlated with NDF and ADF. Fermentation in the rumen of structural carbohydrates, as compared to the fermentation of starch, encourages the growth of acetate producing bacterial species and consequently yielded high production of acetate in the rumen [40]. This is in line with the close correlation between the relative NDF in herbage and the ruminal acetate concentration. Propionate is the major substrate for gluconeogenesis for the ruminant hosts. The concentration of propionate was enhanced after the fermentation of non-fibrous substances by the rumen microorganisms [41]. This is the agreement with the results of the negative correlation between the NDF in herbage and the propionate concentration as well as the CP in herbage which was closely correlated with the propionate concentration in the rumen.

We used the oral stomach tube method for rumen sample collection, realizing only the planktonic associated microbiota is typically retrieved, however this approach allowed us to sample large groups of yaks across herds. Additionally, due to the potential under sampling of rumen contents (1 sample/yak) in the current study, future studies based on sufficient samplings (different sampling locations and sampling time) are needed to provide more representative profiles of the rumen bacterial community. The current study characterized the rumen bacterial composition in yaks during the phenological period. Microbial population in the rumen play a vital role in the production efficiency and the health of the ruminant host [42]. Our data indicated that the *Bacteroidetes*, *Firmicutes*, and *Actinobacteria* were the dominant phyla in the rumen of yak cows in the different phenological periods, and the relative abundances were significantly changed. The majority of the genera present in the different phenological periods with the relative abundance (RAB) ≥1% were similarly affected by the different phenological periods. These microbes are essential for rumen fermentation [43,44]. The composition of the rumen microorganism population in this study is similar to that of the known bacterial population of yak, and they are mainly dominated by *Bacteroidetes* and *Firmicutes*, regardless of feeding groups [2]. The RAB of the phylum *Bacteroidetes* was significantly higher in the vs. than in the BS and SS and the phylum *Firmicutes* was more abundant in the BS than in the vs. and SS, inferring, the main microbial communities were similar for different phenological periods, but each microbial flora’s proportion was different in the rumen for different phenological periods and would have a different fermentability. The previous study reported that dietary various could have important impacts on rumen bacterial community [45]. Under this study, richness estimates, diversity indices, and the main phyla and genera abundances have been found significantly different among the different phenological periods, this was in agreement with previous reports that when ruminants shifted from high-fiber to high-protein, dramatic shifting in rumen microbial population ecology and diversity were obtained [46]. Rumen microbial diversity of dairy cows during the transition from forage to high-grain diets suggesting that *Proteobacteria* would increase as more grain was introduced [47]. Increasing number of *proteobacteria* in high grain diets indicates increased demand for bacterial species capable of metabolizing newly obtained fermentable carbohydrates [47]. In this study, under the different phenological periods, the relative abundance of *Proteobacteria* increased with increasing herbage CP levels, and significant shifting was detected. Our results of remarkable shifting in the abundance of the most phyla could be attributed by chemical compositions of the herbage, suggesting that the rumen bacterial ecology was greatly affected by the herbage nutritive value.

The *Firmicutes* phylum was found as the most abundant, with the value higher than 49% of the total sequences among the different phenological periods and predominantly consisted of *Ruminococcaceae_NK4A214_group* (4.64–7.52%), *Christensenellaceae_R-7_group* (3.84–6.38%), *Butyrivibrio_2* (1.28–2.43%), *Ruminococcaceae_UCG-014* (1.36–2.42%), *Ruminococcaceae_UCG-005* (1.28–2.32%), *Succiniclasticum* (1.07–2.21%), and *Ruminococcaceae_UCG-010* (1.24–2.01%). *Ruminococcaceae_UCG-005* in the rumen is a fiber-digesting bacteria that degrades fiber and is predominantly available in high roughage diets [46]. Under this current experiment, the relative abundance of genus *Ruminococcaceae_UCG-005* in the rumen of the yak was remarkably higher in the SS than in the vs. and BS, *Ruminococcaceae_UCG-005* was higher and closely correlated with the concentration of acetate which will influence the digestion and rumen fermentation. *Butyrivibrio_2* is usually found as fiber-digesting bacteria, it could have added the ability to digest starch and so to produce butyrate [10], while *Butyrivibrio_2* is capable of utilizing fibrous and starchy substrates to synthesis butyrate. This result was in good agreement with the findings of the close correlations between the abundance of *Butyrivibrio_2* and the butyrate concentrations. Other previous studies revealed that the abundance of *Butyrivibrio_2* was greatly reduced in cattle on high-concentrate diets [47]. Whilst under our work, the abundance of *Butyrivibrio_2* in the rumen of yak cows was remarkably higher in the BS than in the vs. and SS. As for *Succiniclasticum*, on important starch-digesting bacteria and is capable of producing propionate through succinate production in the randomization pathway [48]. In our study, the relative abundance of *Succiniclasticum* was remarkably higher in the BS than in the vs. and SS, and agreed with the results of *Succiniclasticum* which was enhanced in cows fed high levels of non-structural carbohydrate [49]. Hypertriglyceridemia is a well-established pre-disposing condition for acute pancreatitis, the hypertriglyceridemia-related necrotizing pancreatitis group would decrease the abundance of intestinal *Christensenellaceae_R-7_group* [50], indicating this bacteria group might be good for the intestinal metabolism under normal status. Furthermore, there existed strong positive correlation between *Christensenellaceae_R-7_group* and DMs such as citrulline, lanosterol, and squalene. Thus, the increase in abundance of *Christensenellaceae_R-7_group* in the BS could serve as the beneficial function of herbage at the BS in improving rumen fermentation and their products.

Sequence analysis of the reads from yaks in the phenological period exhibited an impressive number of bacteria in the phylum *Bacteroidetes* (34.24–48.36%). *Prevotella_1* (16.53–23.65%) was the major available genus belonging to Bacteroidetes phyla. *Prevotella_1* species, which were reported to be the most prevalent bacterial functional group in the rumen microbiome consortium, and they are the predominant in the rumen, regardless of diet compositions [10]. It was reported that ruminal genus *Prevotella_1* exhibited the capability to degrade starches, simple sugars, and other non-cellulosic polysaccharides as energy substrates to produce the glucogenic substrate as succinate, as the major fermentation end-products [51]. Whilst, the polysaccharide-degrading *Prevotellaceae* bacteria were the most prevalent in the rumen of cows fed on diet with the highest starch content [52]. Whilst, several *Prevotella* strains are reported to degrade oligosaccharides and hemicellulose [53]. The abundance of *Prevotella_1* was decreased with increased NDF in the diet [46]. Under this experiment, the results were not found that may be due to the fact that *Prevotella_1* species have hemicellulolytic and proteolytic activities [54], were possibly involved in structural carbohydrates, and the protein or peptide in the rumen fermentation process [55]. The direct-fed of *Prevotella_1* to cows significantly enhanced the ruminal ammonia-nitrogen concentrations [56]. This is consistent with our findings of the close correlations between the abundance of *Prevotella_1* and the ammonia-nitrogen concentrations. The abundant diversity of *Prevotella_1* can improve C_3_ concentration and reduced the C_2_:C_3_ in the rumen fermentation [57]. This finding was in agreement with the earlier findings of the close correlations between the abundance of *Prevotella_1* and the propionate.

## 5. Conclusions

Under this study, different phenological periods impacted the composition of milk, rumen microbiome diversity, and rumen fermentation end-products in yaks that grazed naturally without feed supplementation in the alpine meadow of the QTP. Changes in temperature directly affect plant productivity, which in turn affects animal microbiome in the body. Furthermore, the study provided important data regarding the relationship among herbage nutrient content, rumen bacterial composition, and chemical composition in milk of yaks grazing on the QTP and furthermore offers a foundation for studies aimed at improving ruminant rumen microbial diversity and their relationship with productivity. Future studies using metagenomic and metatranscriptomic analysis are needed to determine whether the microbial metabolic pathways or metabolites may contribute to the composition of milk.

## Figures and Tables

**Table 1:** Major species and proportion of herbage in different phenological periods.

Phenological Periods ^1^	Major Species Communities in the Grassland	Above-Ground Biomass/(g/m^2^)	High Quality Herbage/(g/m^2^)	Other Herbage/(g/m^2^)
VS	*Elymus nutans*, *Poa pratensi*, *Kobresia graminifolia*	43.8	28.6	15.1
BS	*Elymus nutans*, *Poa pratensi*, *Kobresia humili*, *Potentill abifurca*, *Saussurea pulchra*, *Ajania tenuifolia*	286.5	163.2	123.3
SS	*Elymus nutans*	121.4	76.4	45.1

^1^ VS: Vegetative stage, BS: Bloom stage, SS: Senescent stage.

**Table 2:** Nutrient composition of herbage in different phenological periods.

Nutrient Levels ^2^	Phenological Periods ^1^, % of DM			SEM ^3^	*p*-Value
	VS	BS	SS		
DM	34.8 ^c^	46.8 ^b^	64.5 ^a^	0.29	0.025
CP	8.8 ^b^	12.8 ^a^	6.7 ^c^	0.44	<0.01
EE	1.3 ^b^	1.8 ^a^	1.3 ^b^	0.04	<0.01
OM	89.1	89.3	88.5	0.21	0.356
NDF	54.1 ^b^	51.2 ^c^	62.3 ^a^	0.84	<0.01
ADF	35.1 ^b^	32.2 ^c^	36.3 ^a^	0.36	<0.01

^a–c^ Means in a row differ significantly (*p* < 0.05) among the phenological periods. ^1^ VS: Vegetative stage, BS: Bloom stage, SS: Senescent stage. ^2^ Nutrient levels were measured values. DM: dry matter, CP: crude protein, EE: ether extract, OM: organic matter, NDF: neutral detergent fiber, ADF: acid detergent fiber. ^3^ SEM: standard error of the mean.

**Table 3:** Composition of yak milk in different phenological periods.

Component	Phenological Periods ^1^			SEM ^2^	*p*-Value
	VS	BS	SS		
Milk yield, kg/d	1.5 ^b^	2.4 ^a^	1.1 ^c^	0.03	<0.01
Fat, g/100 g	6.2 ^b^	5.4 ^b^	6.7 ^a^	0.10	<0.01
Protein, g/100 g	5.7 ^a^	5.7 ^a^	4.4 ^b^	0.18	<0.01
Lactose, g/100 g	5.5 ^b^	5.9 ^a^	4.8 ^c^	0.10	0.026
Total solids, g/100 g	17.7 ^a^	14.5 ^b^	17.8 ^a^	0.25	0.030

^a–c^ Means within rows are significantly different from each other (*p* < 0.05). ^1^ VS: Vegetative stage, BS: Bloom stage, SS: Senescent stage. ^2^ SEM: standard error of the mean.

**Table 4:** Effects of herbage in different phenological periods on ruminal fermentation of yaks.

Item	Phenological Periods ^1^			SEM ^2^	*p*-Value
	VS	BS	SS		
pH	7.4 ^a^	7.3 ^b^	7.4 ^a^	0.04	0.028
Ammonia N, mg/L	58.4 ^b^	96.5 ^a^	61.4 ^b^	9.18	<0.01
Concentration, mmol/L					
Total VFA ^3^	47.3 ^a^	49.4 ^a^	38.6 ^b^	1.55	<0.01
Acetate	36.2 ^a^	35.5 ^a^	29.5 ^b^	1.08	0.021
Propionate	5.3 ^b^	7.5 ^a^	4.3 ^c^	0.23	<0.01
Butyrate	4.6 ^a^	4.6 ^a^	3.2 ^b^	0.16	0.003
Isobutyrate	0.5 ^b^	0.7 ^a^	0.3 ^c^	0.02	<0.01
Valerate	0.3 ^b^	0.4 ^a^	0.3 ^b^	0.01	<0.01
Isovalerate	0.8 ^a^	0.6 ^c^	0.7 ^b^	0.02	<0.01
Molar proportion, %					
Acetate	76.3 ^a^	71.9 ^b^	76.6 ^a^	0.46	0.036
Propionate	11.5 ^b^	15.3 ^a^	11.3 ^b^	0.16	0.014
Butyrate	9.8	9.3	8.2	0.29	0.072
Isobutyrate	1.1 ^b^	1.4 ^a^	0.7 ^c^	0.02	<0.01
Valerate	0.6	0.8	0.7	0.03	0.206
Isovalerate	1.6 ^b^	1.2 ^c^	1.8 ^a^	0.02	<0.01
Acetate/propionate	6.9 ^a^	4.6 ^b^	6.9 ^a^	0.25	0.022

^a–c^ Means in a row differ significantly (*p* < 0.05) among the phenological periods. ^1^ VS: Vegetative stage, BS: Bloom stage, SS: Senescent stag. ^2^ SEM: standard error of the mean. ^3^ total VFA: total volatile fatty acids.

**Table 5:** Comparison of rumen bacterial phylum and genera ^1^ in different phenological periods.

Bacterial Taxa	Phenological Periods ^2^, %			SEM ^3^	*p*-Value
	VS	BS	SS		
Phylum level					
*Synergistetes*	1.35 ^b^	1.21 ^c^	1.48 ^a^	0.01	<0.01
*Proteobacteria*	1.48 ^b^	1.74 ^a^	1.13 ^c^	0.04	0.009
*Actinobacteria*	2.42 ^a^	1.37 ^c^	1.72 ^b^	0.11	<0.01
*Tenericutes*	1.46 ^b^	2.41 ^a^	2.68 ^a^	0.10	<0.01
*Firmicutes*	43.25 ^b^	57.36 ^a^	46.53 ^b^	1.84	0.023
*Bacteroidetes*	48.36 ^a^	34.24 ^c^	43.73 ^b^	1.78	0.018
Genus level					
*Firmicutes*					
*Christensenellaceae_R-7_group*	4.56 ^b^	6.38 ^a^	3.84 ^b^	0.80	0.035
*Ruminococcaceae_NK4A214_group*	4.64 ^b^	7.52 ^a^	4.83 ^b^	0.39	<0.01
*Ruminococcaceae_UCG-010*	1.38 ^b^	1.24 ^b^	2.01 ^a^	0.07	<0.01
*Butyrivibrio_2*	1.63 ^b^	2.43 ^a^	1.28 ^c^	0.14	0.028
*Ruminococcaceae_UCG-005*	1.28 ^b^	1.41 ^b^	2.32 ^a^	0.09	<0.01
*Eubacterium_coprostanoligenes_group*	0.73 ^c^	1.25 ^b^	1.64 ^a^	0.07	<0.01
*Ruminococcaceae_UCG-014*	1.36 ^b^	2.26 ^a^	2.42 ^a^	0.06	<0.01
*Succiniclasticum*	1.24 ^b^	2.21 ^a^	1.07 ^b^	0.08	0.005
*Actinobacteria*					
*Olsenella*	0.93 ^a^	0.76 ^b^	1.03 ^a^	0.05	<0.01
*Senegalimassilia*	1.84 ^a^	1.31 ^b^	1.22 ^b^	0.03	0.026
*Proteobacteria*					
*Desulfovibrio*	0.58 ^b^	1.06 ^a^	0.14 ^c^	0.01	<0.01
*Bacteroidetes*					
*Prevotella_1*	19.42 ^b^	16.53 ^c^	23.65 ^a^	1.21	0.024
*Rikenellaceae_RC9_gut_group*	7.12 ^b^	6.72 ^b^	9.14 ^a^	0.16	<0.01
*Prevotellaceae_UCG-003*	1.57 ^c^	2.43 ^b^	3.33 ^a^	0.15	<0.01
*Prevotellaceae_UCG-001*	3.23 ^a^	1.84 ^c^	2.20 ^b^	0.15	0.022

^a–c^ Means followed by different letters in a row differ significantly (*p* < 0.05) among the phenological periods. ^1^ Bacterial phylum and genera with relative abundance greater than 1% in at least 60% of yaks within each group were regarded as detected and used for the comparison. ^2^ VS: Vegetative stage, BS: Bloom stage, SS: Senescent stage. ^3^ SEM: standard error of the mean.

## References

[1] Xia W., Osorio J.S., Yang Y., Liu D., Jiang M.F. (2018). Short communication: Characterization of gene expression profiles related to yak milk protein synthesis during the lactation cycle. J. Dairy Sci..

[2] Wang T., Guo Z., Liu Z., Feng Q., Wang X., Tian Q., Ren F., Mao X. (2016). The aggregation behavior and interactions of yak milk protein under thermal treatment. J. Dairy Sci..

[3] Sun W., Luo Y., Wang D.H., Kothapalli K.S., Brenna T. (2019). Branched chain fatty acid composition of yak milk and manure during full-lactation and half-lactation. Prostaglandins Leukot. Essent. Fat. Acids.

[4] Shi B., Jiang Y., Chen Y., Zhao Z., Zhou H., Luo Y., Hu J., Hickford J. (2019). Variation in the Fatty Acid Synthase Gene (FASN) and Its Association with Milk Traits in Gannan Yaks. Animals.

[5] Cui G., Yuan F., Degen A.A., Liu S., Zhou J., Shang Z., Ding L., Mi J., Wei X., Long R. (2016). Composition of the milk of yaks raised at different altitudes on the Qinghai—Tibetan Plateau. Int. Dairy J..

[6] Fan Q., Wanapat M., Hou F. (2019). Mineral Nutritional Status of Yaks (*Bos Grunniens*) Grazing on the Qinghai-Tibetan Plateau. Animals.

[7] Wallace R.J., Sasson G., Garnsworthy P., Tapio I., Gregson E., Bani P., Huhtanen P., Bayat A.R., Strozzi F., Biscarini F. (2019). A heritable subset of the core rumen microbiome dictates dairy cow productivity and emissions. Sci. Adv..

[8] Zhou Z., Fang L., Meng Q., Li S., Chai S., Liu S., Schonewille J.T. (2017). Assessment of Ruminal Bacterial and Archaeal Community Structure in Yak (*Bos grunniens*). Front. Microbiol..

[9] Henderson G., Cox F., Ganesh S., Jonker A., Young W., Janssen P.H., Global Rumen Census Collaborators (2015). Rumen microbial community composition varies with diet and host, but a core microbiome is found across a wide geographical range. Sci. Rep..

[10] Bi Y., Zeng S., Zhang R., Diao Q., Tu Y. (2018). Effects of dietary energy levels on rumen bacterial community composition in Holstein heifers under the same forage to concentrate ratio condition. BMC Microbiol..

[11] Guo W., Bi S., Kang J., Zhang Y., Long R., Huang X., Shan M., Anderson R.C. (2018). Bacterial communities related to 3-nitro-1-propionic acid degradation in the rumen of grazing ruminants in the Qinghai-Tibetan Plateau. Anaerobe.

[12] Xue D., Chen H., Chen F., He Y., Zhao C., Zhu D., Zeng L., Li W. (2016). Analysis of the rumen bacteria and methanogenic archaea of yak (*Bos grunniens*) steers grazing on the Qinghai-Tibetan Plateau. Livest. Sci..

[13] AOAC (2002). Official Methods of Analysis of the Association of Official Analytical Chemists International.

[14] National Food Safety Standard of China (2010). Determination of Lactose and Sucrose in Foods for Infants and Young Children Milk and Milk Products: GB 5413.21-2010.

[15] Van Soest P., Robertson J., Lewis B. (1991). Methods for Dietary Fiber, Neutral Detergent Fiber, and Nonstarch Polysaccharides in Relation to Animal Nutrition. J. Dairy Sci..

[16] Shen J., Chai Z., Song L., Liu J., Wu Y. (2012). Insertion depth of oral stomach tubes may affect the fermentation parameters of ruminal fluid collected in dairy cows. J. Dairy Sci..

[17] Ramos-Morales E., Arco-Pérez A., Martín-García A., Yáñez-Ruiz D.R., Frutos P., Hervás G. (2014). Use of stomach tubing as an alternative to rumen cannulation to study ruminal fermentation and microbiota in sheep and goats. Anim. Feed Sci. Technol..

[18] Chaney A.L., Marbach E.P. (1962). Modified Reagents for Determination of Urea and Ammonia. Clin. Chem..

[19] Yu Z., Morrison M. (2004). Improved extraction of PCR-quality community DNA from digesta and fecal samples. BioTechniques.

[20] Dennis K.L., Wang Y., Blatner N.R., Wang S., Saadalla A., Trudeau E., Roers A., Weaver C.T., Lee J.J., Gilbert J.A. (2013). Adenomatous polyps are driven by microbe-instigated focal inflammation and are controlled by IL-10-producing T cells. Cancer Res..

[21] Magoč T., Salzberg S.L. (2011). FLASH: Fast length adjustment of short reads to improve genome assemblies. Bioinformatics.

[22] Caporaso J.G., Kuczynski J., Stombaugh J., Bittinger K., Bushman F. (2010). QIIME allows integration and analysis of high-throughput community sequencing data. Nat. Methods.

[23] Edgar R.C., Haas B.J., Clemente J.C., Quince C., Knight R. (2011). UCHIME improves sensitivity and speed of chimera detection. Bioinformatics.

[24] Edgar R.C. (2013). UPARSE: Highly accurate OTU sequences from microbial amplicon reads. Nat. Methods.

[25] Amato K.R., Yeoman C.J., Kent A., Righini N., Carbonero F., Estrada A., Gaskins H.R., Stumpf R.M., Yıldırım S., Torralba M. (2013). Habitat degradation impacts black howler monkey (*Alouatta pigra*) gastrointestinal microbiomes. ISME J..

[26] Buxton D.R. (1996). Quality-related characteristics of forages as influenced by plant environment and agronomic factors. Anim. Feed Sci. Technol..

[27] Li H., Ma Y., Li Q., Wang J., Cheng J., Xue S.J., Shi J. (2011). The Chemical Composition and Nitrogen Distribution of Chinese Yak (Maiwa) Milk. Int. J. Mol. Sci..

[28] Liu H., Ren F., Jiang L., Ma Z., Qiao H., Zeng S., Gan B., Guo H. (2011). Short communication: Fatty acid profile of yak milk from the Qinghai-Tibetan Plateau in different seasons and for different parities. J. Dairy Sci..

[29] Grummer R.R. (1991). Effect of Feed on the Composition of Milk Fat. J. Dairy Sci..

[30] Dijkstra J., Ellis J.L., Kebreab E., Strathe A., López S., France J., Bannink A. (2012). Ruminal pH regulation and nutritional consequences of low pH. Anim. Feed Sci. Technol..

[31] Weder C.E., DelCurto T., Svejcar T., Jaeger J., Bailey R.K. (1999). Influence of supplemental alfalfa quality on the intake, use, and subsequent performance of beef cattle consuming low-quality roughages. J. Anim. Sci..

[32] Nafikov R., Beitz D. (2007). Carbohydrate and Lipid Metabolism in Farm Animals. J. Nutr..

[33] Decuypere J.A., Dierick N.A. (2003). The combined use of triacylglycerols containing medium-chain fatty acids and exogenous lipolytic enzymes as an alternative to in-feed antibiotics in piglets: Concept, possibilities and limitations. An overview. Nutr. Res. Rev..

[34] Russell J.B., O’Connor J.D., Fox D.G., Van Soest P.J., Sniffen C.J. (1992). A net carbohydrate and protein system for evaluating cattle diets: I. Ruminal fermentation. J. Anim. Sci..

[35] Grummer R., Clark J., Davis C., Murphy M. (1984). Effect of Ruminal Ammonia-Nitrogen Concentration on Protein Degradation in Situ. J. Dairy Sci..

[36] Lv X., Chai J., Diao Q., Huang W., Zhuang Y., Zhang N. (2019). The Signature Microbiota Drive Rumen Function Shifts in Goat Kids Introduced to Solid Diet Regimes. Microorganisms.

[37] Zhao F., Wang F., King M., Wang L. (2019). Effect of dynamic and static loading during in vitro degradation of a braided composite bioresorbable cardiovascular stent. Mater. Lett..

[38] Suarez-Mena F., Heinrichs A., Jones C., Hill T., Quigley J. (2015). Straw particle size in calf starters: Effects on digestive system development and rumen fermentation. J. Dairy Sci..

[39] Shen H., Lu Z., Xu Z., Chen Z., Shen Z. (2017). Associations among dietary non-fiber carbohydrate, ruminal microbiota and epithelium G-protein-coupled receptor, and histone deacetylase regulations in goats. Microbiome.

[40] Dijkstra J. (1994). Production and absorption of volatile fatty acids in the rumen. Livest. Prod. Sci..

[41] Evans N.J., Brown J.M., Murray R.D., Getty B., Birtles R.J., Hart C.A., Carter S. (2010). Characterization of Novel Bovine Gastrointestinal Tract Treponema Isolates and Comparison with Bovine Digital Dermatitis Treponemes. Appl. Environ. Microbiol..

[42] Goad D.W., Nagaraja T.G. (1998). Ruminal microbial and fermentative changes associated with experimentally induced subacute acidosis in steers. J. Anim. Sci..

[43] Jolazadeh A., Dehghan-Banadaky M., Rezayazdi K. (2015). Effects of soybean meal treated with tannins extracted from pistachio hulls on performance, ruminal fermentation, blood metabolites and nutrient digestion of Holstein bulls. Anim. Feed Sci. Technol..

[44] Wetzels S., Mann E., Metzler-Zebeli B., Wagner M., Klevenhusen F., Zebeli Q., Schmitz-Esser S. (2015). Pyrosequencing reveals shifts in the bacterial epimural community relative to dietary concentrate amount in goats. J. Dairy Sci..

[45] Huws S.A., Kim E.J., Cameron S., Girdwood S.E., Davies L., Tweed J., Vallin H., Scollan N.D. (2014). Characterization of the rumen lipidome and microbiome of steers fed a diet supplemented with flax and echium oil. Microb. Biotechnol..

[46] Tajima K., Aminov R., Nagamine T., Matsui H., Nakamura M., Benno Y. (2001). Diet-Dependent Shifts in the Bacterial Population of the Rumen Revealed with Real-Time PCR. Appl. Environ. Microbiol..

[47] Fernando S.C., Purvis H.T., Najar F.Z., Sukharnikov L.O., Krehbiel C.R., Nagaraja T.G., Roe B.A., DeSilva U. (2010). Rumen Microbial Population Dynamics during Adaptation to a High-Grain Diet. Appl. Environ. Microbiol..

[48] Pope P.B., Smith W., Denman S., Tringe S.G., Barry K., Hugenholtz P., McSweeney C., McHardy A.C., Morrison M. (2011). Isolation of Succinivibrionaceae Implicated in Low Methane Emissions from Tammar Wallabies. Science.

[49] Li R.W., Wu S., Baldwin R.L., Li W., Li C.-J. (2012). Perturbation Dynamics of the Rumen Microbiota in Response to Exogenous Butyrate. PLoS ONE.

[50] Chen J., Huang C., Wang J., Zhou H., Lu Y., Lou L., Zheng J., Tian L., Wang X., Cao Z. (2017). Dysbiosis of intestinal microbiota and decrease in paneth cell antimicrobial peptide level during acute necrotizing pancreatitis in rats. PLoS ONE.

[51] Purushe J., Fouts D.E., Morrison M., White B.A., Mackie R.I., Coutinho P.M., Henrissat B., Nelson W.C., North American Consortium for Rumen Bacteria (2010). Comparative Genome Analysis of Prevotella ruminicola and Prevotella bryantii: Insights into Their Environmental Niche. Microb. Ecol..

[52] Thoetkiattikul H., Mhuantong W., Laothanachareon T., Tangphatsornruang S., Pattarajinda V., Eurwilaichitr L., Champreda V. (2013). Comparative Analysis of Microbial Profiles in Cow Rumen Fed with Different Dietary Fiber by Tagged 16S rRNA Gene Pyrosequencing. Curr. Microbiol..

[53] Emerson E.L., Weimer P.J. (2017). Fermentation of model hemicelluloses by Prevotella strains and Butyrivibrio fibrisolvens in pure culture and in ruminal enrichment cultures. Appl. Microbiol. Biotechnol..

[54] Matsui H., Ogata K., Tajima K., Nakamura M., Nagamine T., Aminov R., Benno Y. (2000). Phenotypic characterization of polysaccharidases produced by four Prevotella type strains. Curr. Microbiol..

[55] Matsui H., Ushida K., Miyazaki K., Kojima Y. (1998). Use of ratio of digested xylan to digested cellulose (X/C) as an index of fiber digestion in plant cell-wall material by ruminal microorganisms. Anim. Feed Sci. Technol..

[56] Chiquette J., Allison M., Rasmussen M. (2008). Prevotella bryantii 25A Used as a Probiotic in Early-Lactation Dairy Cows: Effect on Ruminal Fermentation Characteristics, Milk Production, and Milk Composition. J. Dairy Sci..

[57] Russell J.B., Hespell R.B. (1981). Microbial Rumen Fermentation. J. Dairy Sci..

